# Relationship between Rectal Temperature and Vaginal Temperature in Grazing *Bos taurus* Heifers

**DOI:** 10.3390/ani8090156

**Published:** 2018-09-18

**Authors:** Angela M. Lees, Jim M. Lea, Hannah E. Salvin, Linda M. Cafe, Ian G. Colditz, Caroline Lee

**Affiliations:** 1 CSIRO, Agriculture and Food, Animal Behaviour and Welfare, FD McMaster Laboratory, Armidale, NSW 2350, Australia; jim.lea@csiro.au (J.M.L.); ian.colditz@csiro.au (I.G.C.); caroline.lee@csiro.au (C.L.); 2 DPI, Livestock Industries Centre, Armidale, NSW 2351, Australia; hannah.salvin@dpi.nsw.gov.au (H.E.S.); linda.cafe@dpi.nsw.gov.au (L.M.C.)

**Keywords:** body temperature, cattle, iButton data logger, rectal temperature, vaginal temperature

## Abstract

**Simple Summary:**

Body temperature is widely used to evaluate health status and thermal balance in cattle. There are numerous well-documented measures of body temperature in cattle including rectal, vaginal, tympanic, and rumen. However, in many instances, the relationship that exists between these measures has not been extensively evaluated. This study evaluated the relationship between rectal temperature and vaginal temperature in grazing beef heifers. Gaining a greater understanding of the relationships that exists between measures of body temperature may allow for greater between-study comparisons to occur.

**Abstract:**

This study evaluated the relationship between rectal temperature (T_REC_, °C) and vaginal temperature (T_VAG_, °C) in grazing *Bos taurus* heifers, to develop an understanding of the reliability of these measures as estimates of core body temperature. Nineteen Angus heifers (BW = 232.2 ± 6.91 kg) were implanted with intra-rectal and intra-vaginal data loggers. Rectal temperature and T_VAG_ were simultaneously recorded at 20 s intervals over 18.5 h. Heifers were housed as a singular cohort on grazing pastures for the duration of the study. A strong linear relationship (R^2^ = 0.72, *p* < 0.0001) between the measurement sites was identified. The mean difference between T_REC_ and T_VAG_ was small, in which T_VAG_ was on average 0.22 ± 0.01 °C lower than T_REC_. Individual twenty second T_REC_ and T_VAG_ data were used to determine the pooled mean T_REC_ and T_VAG_ and then to highlight the within measure variation over time. The coefficient of variation was, on average, lower (*p* < 0.001) for T_VAG_ (0.38%) than T_REC_ (0.44%), indicating that T_VAG_ exhibited less variation. Overall, the results from the current study suggest that a strong relationship exists between T_REC_ and T_VAG,_ and that T_VAG_ may be a more reliable estimate of core body temperature than T_REC_ in grazing *Bos taurus* heifers.

## 1. Introduction

Measurements of core body temperature are considered to be a reliable indicator of health status [1], thermal balance [2,3,4], and stress-induced hyperthermia [5,6]. However, providing a precise definition of core body temperature is difficult, as a consistent definition is not available [2]. Traditionally, rectal temperature (T_REC_, °C) has been considered the best estimate of core body temperature. For veterinary clinical examination and field assessment by commercial producers, the measurement of T_REC_ is common practice due to the availability of cost-effective equipment and a simple technique that provides a reliable estimate of body temperature [7]. There are numerous well-documented estimated measures of core body temperature in bovines including tympanic [4,8]; abdominal [3,9], vaginal [10,11], rumen [2,12], and rectal [13,14]. However, in many instances the relationships between the various measures of body temperature have not been comprehensively evaluated. For methods of evaluating body temperature to be considered reliable, a strong association with other validated measures of body temperature is necessary [15,16]. Furthermore, understanding the relationships that exist between the various measures of body temperature may allow for greater between-study comparisons to occur. 

Previous studies have established moderate to strong relationships between T_REC_ and vaginal temperature (T_VAG_, °C) in dairy cows [17,18,19,20] and Brahman heifers [21]. However, previous evaluations of the relationship between different measures of body temperature have often utilized continuous recordings of one measure, i.e., vaginal, compared with time point sampling of another measure, i.e., rectal [2,15,17,18,19,20]. Therefore, these studies may be under or over estimating the relationship that exists between these measures of body temperature. Additionally, the relationship between T_REC_ and T_VAG_ in grazing *Bos taurus* cattle has not been determined. The objective of this study was to evaluate the relationship between T_REC_ and T_VAG_ in grazing Angus (*Bos taurus*) heifers, using a concurrent data capture technique.

## 2. Materials and Methods

This study was conducted with the approval of the CSIRO McMaster Laboratory Animal Ethics Committee (ARA 18-04). The study was undertaken in the New England district of New South Wales, Australia (30.52° S, 151.67° E, 1050 m above mean sea level) at the FD McMaster Research Laboratory. The study was conducted during a southern hemisphere autumn (May). Climatic conditions were monitored at 1 h intervals using an automated weather station (Vaisala Weather Transmitter WXT5200, Vaisaa Oyj, Helsinki, Finland). Average ambient temperature, relative humidity, and wind speed were 13.8 ± 1.07 °C, 45.9 ± 2.75%, and 5.9 ± 0.31 m/s, respectively.

### 2.1. Animals

Nineteen purebred Angus heifers aged between 6.5 and 9.5 months of age, with a mean initial non-fasted live weight of 232.2 ± 6.91 kg, were used in the study. Heifers were weaned 8 weeks prior to the study. Prior to the commencement of the study, heifers were group housed on grazing pastures (*Phalaris aquatica*, *Dactylis glomerata*, and *Plantago lanceolata*) and were supplemented with whole cotton seed. 

### 2.2. Body Temperature

**Figure 1:**
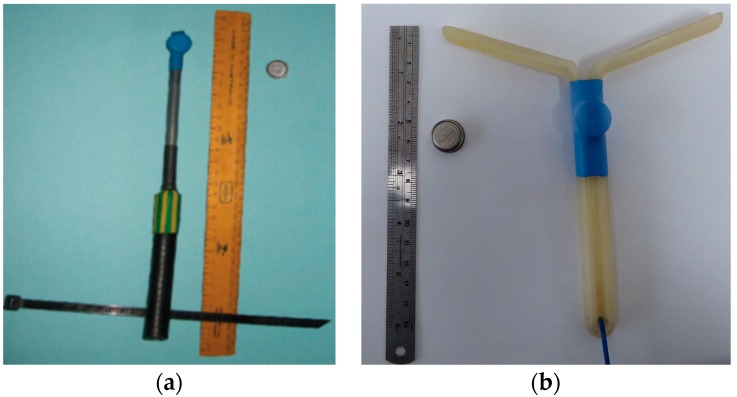
Design of the (**a**) **i**ntra-rectal data loggers as described by Lea et al. [22] and (**b**) **i**ntra-vaginal data loggers.

Rectal temperature and T_VAG_ were recorded at 20 s intervals (iButton DS1922L, Thermochron iButton Device; Maxim Integrated, San Jose, CA, USA). Intra-vaginal and intra-rectal loggers were prepared using a technique modified from Lea et al. [22]. Briefly, intra-rectal loggers consisted of an iButton attached to soft polyethylene piping (180 mm in length × 8 mm in diameter; Figure 1a) and fixed in place using heat shrink plastic. The loggers were inserted into the rectum and held in place using veterinary tape (Tensoplast^®^ Vet, BSN Medical Inc., Hamburg, Germany) to attach the exposed end of the logger to the underside of the tail. For T_VAG_, iButtons were mounted on a progesterone-free controlled internal drug release device (CIDR; 14 cm × 1 cm with a wing span of 15 cm; InterAg New Zealand, Hamilton, New Zealand; Figure 1b). The logger unit was then inserted approximately 20 cm into the vaginal cavity, as described by Verwoerd et al. [23]. Heifers were brought into the handling facilities on day 0 at 1000 h, in which data loggers were placed into the rectal and vaginal cavities. After data loggers were in place, heifers were returned to grazing pastures. Data loggers were programed to commence data collection at 20 s intervals from 1000 h on the following day (day 1). Data loggers remained active for 18.5 h, between 1000 h and 0430 h. Heifers were brought into the handling facilities on day 2 at 0900 h, and data loggers were removed.

### 2.3. Statistical Analysis

One intra-rectal data logger malfunctioned and failed to provide data, another intra-rectal logger was expelled, and the corresponding T_VAG_ data were excluded. Thus, T_REC_ and corresponding T_VAG_ data from 17 animals were analyzed. A linear regression was conducted to determine the coefficient of determination (R, R Foundation for Statistical Computing, Vienna, Austria). To determine accuracy of the dataset, T_REC_ and T_VAG_ data points were matched (with reference to animal ID and time) and directly compared. As there is no precise methodology for determining the true value of core body temperature, T_REC_ and T_VAG_ are both estimated measures of core body temperature, and a relationship between these two measures was anticipated. To evaluate the agreement between T_REC_ and T_VAG_ as estimates of core body temperature, a Bland-Altman plot was constructed [24]. The Bland-Altman plot was constructed by comparing the difference between T_REC_ and T_VAG_ (T_VAG_ minus T_REC_) against the mean of T_REC_ and T_VAG_ [24]. The mean of T_REC_ and T_VAG_ was used as the best functional estimate of core body temperature. Confidence intervals (95%) were added to the Bland-Altman plot to highlight the spread of data. The precision of T_REC_ and T_VAG_ as estimates of core body temperature was determined by evaluating the coefficient of variation at the two measurement sites for each time point (n = 3330). Coefficient of variation values were not normally distributed and were analyzed using a Wilcoxon Signed Rank Test (SigmaPlot, Systat Software Inc., San Jose, CA, USA).

## 3. Results

**Figure 2:**
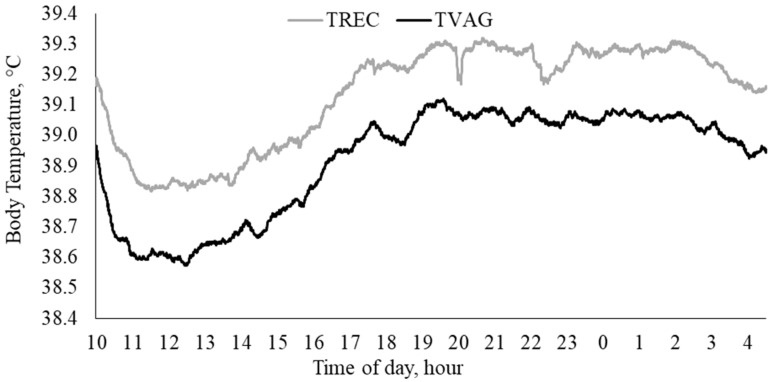
Trend in rectal temperature (T_REC_, °C) and vaginal temperature (T_VAG_, °C) over 18.5 h, in which data were recorded at twenty second intervals.

**Figure 3:**
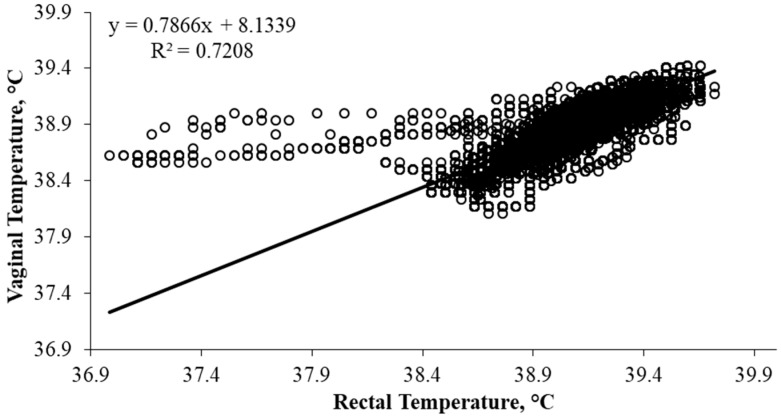
Linear relationship between rectal temperate (T_REC_, °C) and vaginal temperature (T_VAG_, °C) using data recorded at twenty second intervals.

**Figure 4:**
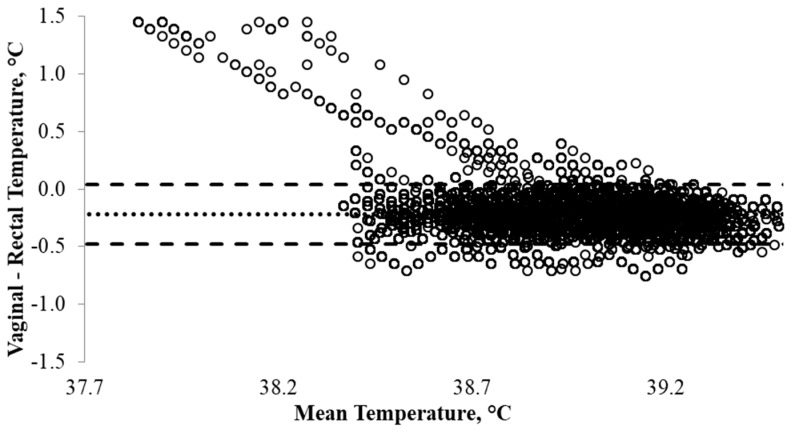
Bland Altman plot assessing the level of agreement between rectal temperature (T_REC_, °C) and vaginal temperature (T_VAG_, °C) recorded at the same time point and the mean difference (dotted line) and confidence intervals (95% = mean ± 1.96 × SD; dashed line). The *x*-axis represents the mean temperature measurement as determined by averaging rectal temperature (T_REC_, °C) and vaginal temperature (T_VAG_, °C), whilst the *y*-axis shows the difference in recorded temperatures for the two methods, in this instance vaginal temperature minus rectal temperature.

Individual twenty second T_REC_ and T_VAG_ data were used to determine pooled mean T_REC_ and T_VAG_ at each time point, and to establish whether a similar pattern existed (Figure 2). The coefficient of determination indicated that there was a strong linear relationship (R^2^ = 0.72, *p* < 0.0001; Figure 3). The Bland-Altman comparison method suggested that the mean difference between T_REC_ and T_VAG_ was small, in which T_VAG_ was, on average, 0.22 ± 0.01 °C lower than T_REC_ (Figure 4). The 95% confidence interval ranged from −0.48 °C to 0.04 °C (Figure 4). The coefficient of variation was on average lower (*p* < 0.001) for T_VAG_ (0.38%) than T_REC_ (0.44%). 

## 4. Discussion

Rectal and vaginal temperatures appeared to follow a similar pattern over the duration of the study (Figure 2). Vaginal temperatures were consistently lower than T_REC_ and did not appear to have rapid temperature fluctuations (Figure 2). This suggests that T_VAG_ may be less sensitive to temperature changes influenced by other factors, particularly defecation. Therefore, T_VAG_ may be a better reflection of changes in core body temperature, providing a more reliable measure of body temperature. Furthermore, the vaginal cavity is likely to have a greater blood flow compared with the rectum and consequently may be more sensitive to core temperature changes [21]. Lower than expected T_RECs_ (≤37.5 °C) were observed in one heifer, in which these data points are easily identified in Figure 3 and Figure 4. These data were not excluded from the data set as they were considered to be within a physiologically acceptable range (≥37.0 °C). Furthermore, whilst these T_REC_ data points were ≤37.5 °C, the corresponding T_VAG_ were ≥38.5 °C, suggesting that the low T_REC_ occurred as a result of displacement of the rectal probe. This displacement may have occurred as the animal transitioned into a lying position, repositioning the intra-rectal data logger and/or causing air infiltration into the rectal cavity. This is supported by Burfeind et al. [25], concluding that T_REC_ was 0.4 ± 0.2 °C greater (*p* < 0.001) when the thermometer was placed deeper in the rectum (6 cm versus 11.5 cm). Furthermore, excluding these T_REC_ (≤37.5 °C) and the corresponding T_VAG_ data had a limited influence on the relationship (R^2^ = 0.78, *p* < 0.0001), and the mean difference between T_REC_ and T_VAG_ was negligible (0.23 ± 0.01 °C). 

A strong relationship between T_REC_ and T_VAG_ was observed within the current study (R^2^ = 0.72; *p* < 0.0001). The coefficient of variation was on average lower (*p* < 0.001) for T_VAG_ than T_REC_, suggesting that there was less variation in T_VAG_ in the current study. Previous studies have suggested that a strong relationship exists between T_REC_ and T_VAG_ in *Bos indicus* heifers (r = 0.92, *p* < 0.0001; Burdick et al. [21]), pregnant dairy cows (R^2^ = 0.90, *p* < 0.05; Hillman et al. [19]), and lactating dairy cows (r = 0.81, *p* < 0.001, Vickers et al. [18]; r = 0.92 ≤ 0.94, *p* < 0.001, [26]), although the strength of the relationship between T_REC_ and T_VAG_ decreased (r = 0.46, *p* < 0.001) during peak lactation [18]. Additionally Kaufman et al. [20] showed that the relationship between T_REC_ and T_VAG_ increased from morning (1000 h, r = 0.47, *p* < 0.01) to afternoon (1500 h, r = 0.69, *p* < 0.01) in lactating dairy cows. The relationships identified within the current study are greater than those described by Hillman et al. [19], Vickers et al. [18], and Kaufman et al. [20]. However, the relationships between T_REC_ and T_VAG_ within these studies were evaluated using intra-vaginal data loggers and hand-held thermometers to obtain time point measurements of T_REC_, with variable intervals. In the current study and studies by Burdick et al. [21] and Suthar et al. [26], T_REC_ and T_VAG_ were measured concurrently using indwelling temperature data loggers. These results suggest that simultaneous measurements may improve the relationship observed between T_REC_ and T_VAG_. Regardless, the current study is the first to evaluate the relationship between T_REC_ and T_VAG_ in grazing *Bos taurus* heifers, using a simultaneous data capture technique.

Body temperature in many mammalian species has a circadian rhythm in which body temperature is at its lowest during the morning and highest in the evening [2,9,27,28,29]. It is important to consider the impact that the circadian rhythm may have on the relationship between T_REC_ and T_VAG_. Although a circadian rhythm cannot be definitely established within the current study, due to the restricted data collection period, a trend appeared to exist (Figure 1). To effectively define the circadian rhythm in body temperature measurements, longer observation periods are required. Furthermore, climatic conditions may influence the dynamic range of the circadian rhythm [9,28,30], as the variations observed in body temperature are a reflection of the equilibrium between the amount of heat energy produced/accumulated and dissipated from the body [31]. Further studies conducted over longer periods of time and under different climatic conditions are warranted in order to more accurately define the relationship that exists between T_REC_ and T_VAG_. 

When comparing these two measurement methodologies, it is important to recognize that neither methodology may be ideal. Defining core body temperature is difficult, as a consistent definition or measure has not been identified [2]. Thus, numerous measures have been used as an estimation of core body temperature in beef cattle [2,3,8,13], and defining the relationship that exists between measurements becomes difficult. However, the strong relationship observed within the current study suggests that body temperature comparisons between male (T_REC_) and female (T_VAG_) cattle are potentially possible, although further studies are required to determine an appropriate correction factor, to ensure that one measure was not over or under estimating the other measure.

Using correlations and/or regression models to define the relationship between the two measures used to estimate core body temperature may be misleading, as linear and non-linear models do not describe the agreement between two methods of measurement. Rather, they are a measure of association between the two measures [32,33,34]. Given that T_REC_ and T_VAG_ are both estimated measures of core body temperature, it would be unusual if a relationship did not exist. An alternative method of evaluating the agreement between T_REC_ and T_VAG_ may be provided by conducting a Bland-Altman analysis [35]. The Bland-Altman methodology measures the limits of agreement, thus determining whether T_REC_ and T_VAG_ are comparable, and evaluates the degree of agreement between T_REC_ and T_VAG_ (Figure 4) [36]. As body temperature is typically maintained within a small dynamic range, usually within ±1 °C [37], the Bland-Altman method of comparison [24] assesses the relationship between the two measures by using T_VAG_ minus T_REC_. By using the Bland-Altman method, results from the current study indicated that the mean difference between T_REC_ and T_VAG_ was small (0.22 ± 0.01 °C), with a 95% confidence interval of −0.48 °C to 0.04 °C. Overall, these results suggest that T_REC_ and T_VAG_ are comparable; however, the coefficient of variation indicates that T_VAG_ may be a more precise estimate of core body temperature.

The data capture methodologies used during the current study do have some disadvantages. The data loggers were not active radiotelemetry devices; hence, the data were stored and downloaded at the conclusion of the study. Thus, there is the potential for data loggers to corrupt and/or fail within the data collection phase [36], which occurred within the current study, contributing to a data loss of 5.3%. An additional data logger was displaced from the rectal cavity; therefore, the total data loss within this study was 10.5%. Unfortunately, there is no method of determining whether a data logger has become corrupted or failed, until the data download phase [36,38]. An advantage of radiotelemetry devices is that radio transmissions can be communicated and transcribed to a database providing real time measurements of body temperature [2,15]. Nonetheless, data loggers remain a reliable method of obtaining measurements of body temperature within research studies [21,36].

## 5. Conclusions

Rectal temperature and T_VAG_ have been used as a proxy for core body temperature within research studies for numerous years. Advances in technology enabled the current study to be the first to evaluate the relationship between T_REC_ and T_VAG_ in grazing *Bos taurus* heifers, using a simultaneous data capture technique. These data highlighted that a strong relationship exists between T_REC_ and T_VAG_. Furthermore, these results suggest that T_VAG_ may provide a more sensitive and reliable estimate of core body temperature than T_REC_ in grazing *Bos taurus* heifers.
